# Sociodemographic Comparison of Children With High-risk Medical Conditions Referred vs Identified Through Screening Plus Outreach for COVID-19 Therapeutics

**DOI:** 10.1001/jamanetworkopen.2022.48671

**Published:** 2022-12-28

**Authors:** Simon Parzen-Johnson, Shan Sun, Ami B. Patel, Tonya L. Scardina, Seema K. Shah, Sameer J. Patel

**Affiliations:** 1 Division of Infectious Diseases, Ann and Robert H. Lurie Children’s Hospital of Chicago, Chicago, Illinois; 2 Northwestern University, Feinberg School of Medicine, Chicago, Illinois; 3 Department of Pharmacy, Ann and Robert H. Lurie Children’s Hospital of Chicago, Chicago, Illinois; 4 Division of Advanced General Pediatrics, Ann and Robert H. Lurie Children’s Hospital of Chicago, Chicago, Illinois

## Abstract

**Importance:**

Minoritized groups are less likely to receive COVID-19 therapeutics, but few studies have identified potential methods to reduce disparities.

**Objective:**

To determine whether screening plus outreach, when compared with referral alone, increases identification of vulnerable pediatric patients at high risk for severe disease eligible for COVID-19 therapeutics from low-resourced communities.

**Design, Setting, and Participants:**

A retrospective cohort study of COVID-19 medication allocation between January 1, 2022, and February 15, 2022, at Lurie Children’s Hospital, a quaternary care children’s hospital, in Chicago, Illinois. The cohorts were pediatric patients referred for COVID-19 therapeutics or with a positive SARS-CoV-2 polymerase chain reaction within the hospital system followed by outreach. Screening involved daily review of positive cases of SARS-CoV-2, followed by medical record review for high-risk conditions, and communication with clinicians and/or patients and families to offer therapy.

**Exposures:**

Diagnosis of COVID-19.

**Main Outcomes and Measures:**

The primary measure was difference in child opportunity index (COI) scores between the 2 cohorts. Secondary measures included presence and duration of symptoms at diagnosis, medication uptake, race and ethnicity, insurance type, qualifying medical condition, sex, primary language, and age.

**Results:**

Of 145 total patients, the median (IQR) age was 15 (13-17) years, and most were male (87 participants [60.0%]), enrolled in public insurance (83 participants [57.2%]), and members of minoritized racial and ethnic groups (103 participants [71.0%]). The most common qualifying conditions were asthma and/or obesity (71 participants [49.0%]). From 9869 SARS-CoV-2 tests performed, 94 eligible patients were identified via screening for COVID-19 therapeutics. Fifty-one patients were identified via referral. Thirty-two patients received medication, of whom 8 (25%) were identified by screening plus outreach alone. Compared with referred patients, patients in the screening plus outreach group were more likely to have moderate, low, or very low COI composite scores (70 patients [74.5%] vs 27 patients [52.9%]); public insurance (65 patients [69.1%] vs 18 patients [35.3%]); and asthma or obesity (60 patients [63.8%] vs 11 patients [21.6%]). Patients in the referral group were more likely to be non-Hispanic White (23 patients [45.1%] vs 19 patients [20.2%]) and receive medication (24 patients [47.1%] vs 8 patients [8.5%]).

**Conclusions and Relevance:**

Compared with referral patients, screening plus outreach patients for COVID-19 medications were more socially vulnerable, with lower COI scores, and more likely to have asthma or obesity. Future studies should investigate communication strategies to improve uptake of these medications after outreach.

## Key Points

**Question:**

Compared with referral, was screening plus outreach associated with identification of more vulnerable children from low-resourced communities with high-risk medical conditions in whom COVID-19 therapeutics were indicated?

**Findings:**

In this cohort study of 145 patients, screening plus outreach was associated with increased numbers of patients offered and receiving medication over referrals alone. Patients identified by screening were more likely to have child opportunity index scores of moderate, low, or very low; public health insurance; asthma and/or obesity; and be from a minoritized racial or ethnic group.

**Meaning:**

These findings suggest that screening plus outreach is associated with increased identification of socially vulnerable at-risk children who qualified for COVID-19 therapeutics.

## Teaser

This cohort study evaluates the sociodemographic differences between children with high-risk medical conditions who were referred vs identified through screening plus outreach for COVID-19 therapeutics.

## Introduction

Medications to treat COVID-19 such as monoclonal antibodies (mAb) and antivirals (remdesivir, nirmatrelvir/ritonavir) are effective in reducing risk of hospitalization and death in high-risk populations.^1^ By December 2021, multiple medications to treat mild to moderate COVID-19 in adults and adolescents were authorized by the Food and Drug Administration (FDA), but supply was limited relative to demand due to the surge in cases caused by the Omicron variant.^2,3,4^ Despite direct distribution by the federal government and local health departments, racial and ethnic disparities in receiving treatments have been documented, along with lower access to mAb for patients living in socially vulnerable communities.^5,6^ Patients with lower socioeconomic status (SES) and less English proficiency have also faced substantial barriers to other COVID-19 mitigation measures, including vaccination, despite their association with higher COVID-19 morbidity and mortality.^7,8^

The National Institutes of Health recommended prioritizing patients at highest risk of severe illness when medication supply is scarce, but evidence to inform risk stratification is limited, particularly in children.^9^ Even if prioritization criteria are well-defined, racial minority groups and lower income families with qualifying chronic conditions may not have equal access to these medications due to information deficits and barriers to care. To mitigate health inequities and prevent harm to patients at high risk, health systems can use indices of disadvantage to prospectively identify patients who would otherwise be less likely to access potentially life-saving therapies.^10^

The standard approach of relying on physician-initiated referrals for COVID-19 medications is dependent on clinicians’ time and knowledge, which may be limited with new therapeutics that have unconventional distribution mechanisms and in clinics with fewer resources. Patient-initiated referrals may also be constrained by information deficits and barriers to access. Given the diverse urban setting of our patient population, we hypothesized that screening plus outreach, when compared with referrals, would be associated with increased administration of COVID-19 medication and would be associated with increased identification of high-risk children with lower SES and associated health conditions.

## Methods

### Intervention Setting

This study is a retrospective analysis of allocation of medications used to treat COVID-19 at the Ann and Robert H. Lurie Children’s Hospital of Chicago, Illinois (Lurie Children’s), from January 1, 2022, to February 15, 2022. All patients associated with the Lurie Children’s hospital system, an urban children’s hospital that serves a population primarily insured through Medicaid, during this period, qualified for inclusion in the study. Lurie Children’s is a quaternary care children’s hospital, serving the metropolitan Chicago area with a population of approximately 9 million people.^11^ Roughly 80 000 visits are made to the emergency department (ED) and urgent care centers each year. There are more than 600 000 outpatient visits per year to 14 outpatient and surgery centers throughout the region. To define general demographic data for patients seen at Lurie Children’s, and within the ED during the study period, demographic variables were assessed for all patients with any outpatient and/or inpatient encounter at Lurie Children’s. This study was deemed exempt from review and the need for informed consent by the local institutional review board because study activities were within the scope of improving health care delivery. Strengthening the Reporting of Observational Studies in Epidemiology (STROBE) reporting guidelines were reviewed and assessed in relation to the data reported in this study.

### COVID-19 Therapy Allocation

Sotrovimab and nirmatrelvir/ritonavir were first available on January 1, 2022, and January 11, 2022, respectively. Criteria for eligibility (eAppendix 1 in Supplement 1) were developed in December 2020, modified from FDA Emergency Use Authorization criteria, with relevant subspecialty consultation.^12^ Due to anticipated medication scarcity, priority tiers were developed in December 2021 (eAppendix 2 in Supplement 1), with input from the Bioethics Program, and a group of 15 other physicians from pediatric subspecialties (infectious diseases, cardiology, pulmonology, oncology, gastroenterology-hepatology, rheumatology, and nephrology). Vaccine status was not a criterion for referral or allocation of medication. Molnupiravir was not offered at Lurie Children’s Hospital.

mAbs were administered either in the ambulatory infusion center or inpatient medical units (with discharge after infusion) Monday through Saturday. On Sunday, infusions were performed only in the inpatient setting. Nirmatrelvir/ritonavir was available via infectious disease clinic visits 5 days per week, or via ED dispense (7 days per week, 24 hours per day).

### Referral

Clinicians were able to refer patients 24 hours per day by contacting the on-call infectious diseases physician or the institutional contact for outpatient COVID-19 therapeutics (S.J.P.). Mechanisms of communication for referral included the paging system, email, Epic in-basket messages, and a Qualtrics online form. The Qualtrics form facilitated referrals in the Chicago metropolitan area who were not part of the hospital network and/or did not share the same electronic health record. S.J.P. served as the contact person for COVID-19 therapeutics and conducted outreach via hospital emails, presentations, and Zoom calls, including education on eligibility and referrals. In addition, parents were able to call the Infectious Diseases office. All patients in the Chicago metropolitan area, even without receipt of care in the Lurie Children’s system, were eligible for referral.

### Screening Plus Outreach

**Figure 1.**
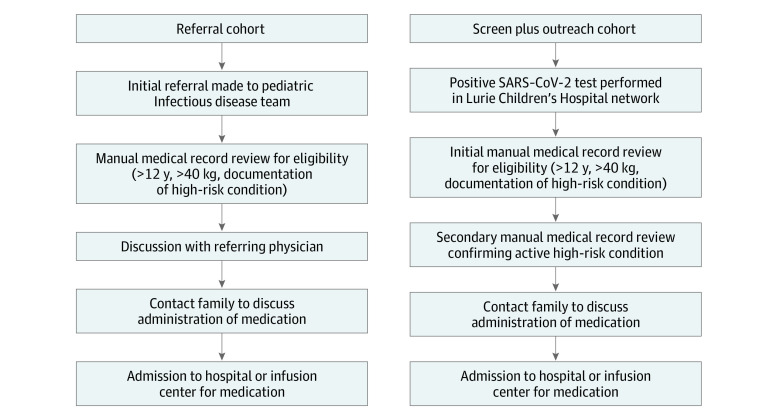
Cohort Identification Flowchart

In addition to referrals, screening followed by outreach was performed (Figure 1). All positive SARS-CoV-2 test results within the Lurie Children’s Hospital System (ED, outpatient, and inpatient) were reviewed daily by a physician in the pediatric infectious diseases department. If patients were aged 12 years old or older and weighed 40 kg or more, their medical records were reviewed for factors associated with risk that may suggest the patient would be eligible for COVID-19 therapy (eAppendix 1 in Supplement 1). If they passed this initial screening process, the patient record was then reviewed by a second physician and if deemed eligible, the patient’s family was contacted to discuss therapeutic options. If there were concerns for availability of medications, or facilities for medication administration, priority was given according to the degree of risk of progression to severe disease (eAppendix 2 in Supplement 1). The priority schema was developed according to expert consensus and existing national recommendations. If patients were deemed eligible according to this screening process, the family was contacted directly to discuss eligibility for therapy. Whether via referral or screening plus outreach, eligibility for COVID-19 therapeutics was assessed on a case-by-case basis with medical record review and/or discussion with the referral clinician. Although asymptomatic patients were referred or screened, only symptomatic patients were offered therapy.

### Review of COVID-19 Medication Allocation

SARS-CoV-2 test results, screening, referrals, and COVID-19 medication allocation were reviewed from January 1, 2022, to February 15, 2022. All eligible patients either had a positive SARS-CoV-2 nasopharyngeal polymerase chain reaction obtained at Lurie Children’s or were referred to the pediatric infectious diseases division with a positive test. Patients were classified into 2 groups: patients identified from referrals or by screening. Patients who were identified by both modalities were placed in the referral cohort for statistical comparison, given that they would have been referred regardless of screening procedures.

For analysis, the 2 cohorts were defined at the step where referral or screening was first initiated (Figure 1). This was deliberately chosen to evaluate the potential limitations of each method to identify eligible patients. Some patients in each cohort were eventually determined to be ineligible because they were asymptomatic, did not meet high risk criteria, or were younger than 12 years or weighed less than 40 kg.

The primary variable of interest was the difference in child opportunity index (COI) between the 2 cohorts. COI is an index of neighborhood features, separated into 3 domains (education, health and environment, social and economic), that are converted to *z* scores weighted according to published economic mobility and health indicators.^13^ The scores for all patients were obtained via cross-referencing patient zip codes with published zip code COI estimates.^13^ When assessing proportional differences between groups, COI scores were grouped into 2 categories, very high or high and moderate, low, or very low. Secondary measures included differences in age, sex, race and ethnicity, preferred language, insurance provider, qualifying diagnosis, presence of symptoms at diagnosis, days of symptoms before diagnosis, and receipt of COVID-19 directed therapy. These variables were all determined according to medical record review using physician documentation. Primary language was categorized as non-English or English. Insurance was categorized as commercial or public. Race and ethnicity were self-reported and were dichotomized as non-Hispanic White vs all other races and ethnicities (non-Hispanic Black, Hispanic, and any other race or ethnicity [other includes Asian, non-Hispanic/Latinx; 2 or more races, non-Hispanic/Latinx; not given]). Race and ethnicity were examined to evaluate disparities in allocation by these parameters, as have previously been shown.^5^ These variables were all abstracted from patient electronic medical records.

### Statistical Analysis

Categorical variables were described with counts and proportions. Continuous variables were described with medians and IQRs. The primary and secondary factors associated with risk were compared between the 2 cohorts using 2-sided χ^2^ tests. Significance was set at *P* = .05. All statistical analyses were conducted using SAS Enterprise Guide statistical software version 7.1 (SAS Institute). All proportions were calculated with nonmissing values.

## Results

### Demographic Characteristics

A total of 145 patients were included in this retrospective cohort study. Most patients were male (87 participants [60.0%]) and enrolled in public insurance (83 participants [57.2%]). The median (IQR) age of patients in this study was 15 (13-17) years. The most common qualifying condition for therapeutics was asthma and/or obesity (71 participants [49.0%]). Overall, 42 participants (29.0%) were non-Hispanic White; 20 (13.8%) were Black, non-Hispanic, 71 (49.0%) were Hispanic/Latinx, and 12 (8.3%) were of other races and ethnicities (103 participants from minoritized groups [71.0%]) (Table 1).

**Table 1.** Demographics, Child Opportunity Index Scores, and Incidence of COVID-19 Treatment Prescribed Among Referred and Screened Patients

Category	Patients, No. (%)^a^		*P* value
	Referral (n = 51)	Screening (n = 94)	
Age, median (IQR), y	15 (13-17)	15 (13-16)	.37
Days of symptoms before medication receipt, median (IQR)	4 (3-5)	2 (1-2)	.08
Presence of symptoms at diagnosis	43 (93.5)	64 (75.3)	.01^b^
Qualifying condition, asthma and/or obesity	11 (21.6)	60 (63.8)	<.001^c^
Received medication	24 (47.1)	8 (8.5)	<.001^c^
Sex			
Female	23 (45.1)	35 (37.2)	.36
Male	28 (54.9)	59 (62.8)	
Race and ethnicity			
Non-Hispanic			
White	23 (45.1)	19 (20.2)	.002^c^
Black	6 (11.8)	14 (14.9)	
Hispanic or Latinx	16 (31.4)	55 (58.5)	
Other^d^	6 (11.8)	6 (6.4)	
Primary language, English	43 (84.3)	70 (74.5)	.17
Public insurance	18 (35.3)	65 (69.1)	<.001^c^
Child Opportunity Index score, moderate, low, or very low			
Composite	27 (52.9)	70 (74.5)	.009^c^
Education	24 (47.1)	67 (71.3)	.004^c^
Health and Environment	33 (64.7)	86 (91.5)	<.001^c^
Social and Economic	26 (51.0)	68 (72.3)	.01^b^

^a^ All proportions were calculated according to nonmissing values.

^b^ *P* < .05.

^c^ *P* < .01.

^d^ Other includes Asian, non-Hispanic or Latinx; 2 or more races, non-Hispanic or Latinx; not given

### Screen Plus Outreach Cohort

**Figure 2.**
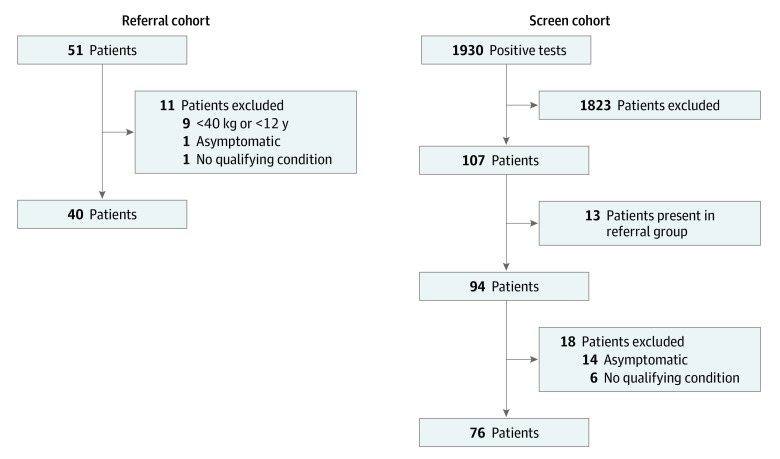
Patients Identified and Excluded at Each Step of Patient Identification

During the study period, a total of 9869 SARS-CoV-2 tests were performed; 2818 (28.6%) tests were performed in the ED, 6252 (63.4%) in outpatient settings, and 799 (8.1%) in the inpatient setting. A total of 1930 patients tested positive (19.6% of total tests) and underwent the screening process. Of positive tests, 692 (35.9%) were from the ED, 1138 (59.0%) were from the outpatient setting, and 100 (5.2%) were from the inpatient setting. After exclusion of 13 patients who were also referred, there were 94 patients who were aged 12 years or older and weighed 40 kg or more who met criteria for COVID-19 therapy on initial screening. Of these 94 patients, 76 (80.9%) were considered eligible following a second physician review (Figure 2). Forty-two patients (44.7%) tested positive in the ED, whereas 52 patients (55.3%) were tested outside of the ED setting. All qualifying conditions for the screening plus outreach and referral cohorts are listed in Table 2. A summary of patients with clinical encounters for all diagnoses during the study period is listed in eAppendix 3 in Supplement 1 for reference.

**Table 2.** Qualifying High-risk Conditions Among Initially Screened and Referred Patients COVID-19 Diagnoses

Condition	Referral (n = 51)	Screening (n = 94)
Solid organ transplant	12 (23.5)	7 (7.5)
Oncology	8 (15.7)	6 (6.4)
Other immunocompromised^a^	7 (13.7)	5 (5.3)
Cardiac	8 (15.7)	6 (6.4)
Obesity	8 (15.7)	30 (31.2)
Asthma	1 (1.96)	20 (21.3)
Asthma and/or obesity	2 (3.9)	10 (10.6)
Other^b^	5 (9.8)	10 (10.6)

^a^ Other immunocompromisec includes combined variable immunodeficiency, DiGeorge syndrome, Goodpasture syndrome, inflammatory bowel disease, juvenile inflammatory arthritis, nephrotic syndrome, systemic lupus erythematosus, tuberous sclerosis.

^b^ Other includes cerebral palsy, chronic lung disease, cystic fibrosis, diabetes, Duchenne muscular dystrophy, Lennox-Gastaut syndrome, neurofibromatosis-1, Niemann-Pick disease, pulmonary hypertension, sickle cell disease, and trisomy-21.

### Referral Cohort

A total of 51 patients were referred for COVID-19 therapy during the study period. Of these patients, 40 (78.4%) were considered eligible for therapy and the patient or their families were contacted to discuss therapy (Figure 2). Thirty-three of the 51 patients (64.7%) were referred via email, 1 (2.0%) via EPIC in-basket messaging, 2 (3.9%) via the paging system, and 15 (29.4%) via the Qualtrics form. Twenty-one patients were referred from general pediatrics (including 6 from outside the Lurie health care system), 8 from oncology or stem cell transplant, 5 from kidney transplant, 4 from cardiac transplant, and 13 from other subspecialty groups (eAppendix 4 in Supplement 1). The median (IQR) age for referred patients was 14 years (12-17).

### Group Comparison

Within the referral cohort, 78.4% of patients (40 of 51) met criteria and were contacted regarding therapy, whereas 47.1% (24 of 51 patients) agreed to and received COVID-19 therapy. Within the screening cohort, 80.9% of patients (76 of 94) met criteria and were contacted regarding therapy, while 8.5% (8 of 94 patients) agreed to and received COVID-19 therapy. Although we did not record reasons for declination for all patients, common reasons given by the parents included perceptions that children were improving without therapy and concerns for potential adverse events with a newly FDA-authorized medication. Compared with referred patients, patients in the screening group (Table 1) were more likely to have moderate, low, or very low composite COI scores (70 patients [74.5%; 95% CI, 65.7%-83.3%] vs 27 patients [52.9%; 95% CI, 39.2%-66.6%]; *P* = .009), as well as in all COI subgroups: education (67 patients [71.3%; 95% CI, 62.1%-80.4%] vs 24 patients [47.1%; 95% CI, 33.4%-60.8%]; *P* = .004), health and environment (86 patients [91.5%; 95% CI, 85.8%-97.1%] vs 33 patients [64.7%; 95% CI, 51.6%-77.8%]; *P* < .001), and social and economic (68 patients [72.3%; 95% CI, 63.3%-81.4%] vs 26 patients [51.0%; 95% CI, 37.3%-64.7%]; *P* = .01). Compared with referred patients, patients in the screening group were more likely to have public health insurance (65 patients [69.1%; 95% CI, 59.8%-78.5%] vs 18 patients [37.5%; 95% CI, 23.8%-51.2%]; *P* < .001); asthma and/or obesity (60 patients [63.8%; 95% CI, 54.1%-73.5%] vs 11 patients [21.6%; 95% CI, 10.3%-32.9%]; *P* < .001), and race and ethnicity other than non-Hispanic White (75 patients [79.8%; 95% CI, 71.7-87.9%] vs 28 patients [54.9%; 95% CI, 41.2%-68.6%]; *P* = .002). Patients in the referral cohort were more likely to be symptomatic when tested (43 patients [93.5%] vs 64 patients [75.3%]; *P* = .01). Patients in the referral cohort had a longer median duration between symptom onset and medication receipt (4 days vs 2 days; *P* = .08), however this was based on limited samples as symptom onset was not documented for 3 of 8 patients treated in the screening plus outreach group. Patients in the referral group were more likely to be White, non-Hispanic (23 patients [45.1%] vs 19 patients [20.2%]; P = .002) and receive medication (24 patients [47.1%] vs 8 patients [8.5%]; P < .001). There were no differences between groups with regard to age at the time of screening, days of symptoms before testing positive, eligibility rate, sex, and primary language. During the period of study, we estimate the screening and referral process to have taken 4 hours per week of physician time each.

Despite potential shortages, there was only 1 patient in the referral cohort who did not receive medication due to supply or scheduling limitations. All patients in the study received sotrovimab except for 2 who received nirmatrelvir/ritonavir.

## Discussion

Newly authorized medications to treat COVID-19 have been a critical resource to decrease hospitalizations and mortality. Screening and outreach led to 33% more patients receiving mAb. This cohort study demonstrates that screening plus outreach is associated with increased receipt of COVID-19 medications by patients in general, as well as for those who have public insurance or are likely to live in communities with less education resources, access to healthy food, and employment resources.^13^ Relying solely on referrals may limit access to these potentially life-saving medications for patients from vulnerable communities disproportionately affected by COVID-19.

Dissemination of scarce resources according to screening and outreach may be an important means to reach patients with certain underlying medical conditions. Patients who were screened were significantly more likely to have asthma or obesity as their qualifying condition (63.8%) than patients who were referred (21.6%). Obesity is more common in adolescents in communities with lower incomes and COI scores but may not be recognized as a factor associated with risk by clinicians or parents, despite risk of progression to severe illness and inclusion in FDA authorization criteria.^14,15^ Furthermore, families from these communities may face barriers to accessing care, including difficulty with English, challenges with obtaining leave from work, and lack of nearby clinics. Conversely, parents of children with other conditions that require frequent follow-up (eg, organ transplant, chronic lung disease) may be more likely to contact clinicians after SARS-CoV-2 infection, and their subspecialty clinicians may be primed to seek infectious disease consultation. Maximizing uptake of COVID-19 medications for all qualifying health conditions is essential to reduce burden of COVID-19 hospitalizations to health care systems, particularly when hospital bed space is limited during COVID-19 surges.

Screening and outreach facilitates access to infectious diseases expertise, which is important for patients with lower SES who access subspecialists less often and may delegate more responsibility to their primary care clinicians.^16^ Although global emails were disseminated and specific primary care clinic engagement was performed to disseminate opportunities to referral, this process could be streamlined in the future. Even when therapy was declined, screening and outreach was an opportunity to engage with families who could benefit from education about COVID-19 mitigation, including measures to reduce household transmission, guidance if symptoms worsened, and recommendations to contact clinicians for COVID-19 therapeutics for high risk family contacts. These results may have implications beyond COVID-19 therapeutics. For example, screening may be beneficial to identify children at high risk for respiratory syncytial virus who might qualify for respiratory syncytial virus immunoprophylaxis. Future studies should examine the costs and benefits of screening and outreach for other pediatric conditions and interventions.

Compared with referrals, patients who were identified by screening were less likely to agree to receipt of medication. There may be multiple reasons for this difference. Patients who were referred may have already agreed to therapy after initial discussion with their clinicians, while patients who were identified by screening may have been learning about COVID-19 medications for the first time. Furthermore, patients with chronic conditions requiring frequent follow-up (ie, organ transplant/oncologic conditions vs asthma/obesity) are likely to have trusting, long-term relationships with their clinical care team, which has been shown to be associated with medication adherence, and likely acceptance of COVID-19 therapeutics.^17^ Another potential explanation may be that families of patients with lower COI could find it challenging to come to our health care facility for mAb infusions. Transportation challenges, travel costs, lack of childcare, or work constraints can all be associated with low percentages of medication receipt.

Although racial and ethnic differences between the 2 groups exist, this alone is unlikely to explain differences in medication receipt. Belonging to a minoritized group has been described to be associated with a lower likelihood of having the intent to vaccinate against SARS-CoV-2.^18,19,20^ Interest in vaccination may be different, however, than interest in receiving treatment after a positive diagnosis. Furthermore, the refusal of medication does not appear to be the main factor associated with racial and ethnic disparities in receipt of medication among adults in our metropolitan area.^6^ Further research into racial and ethnic differences in medication receipt after approach from clinicians is warranted.

### Future Interventions

Future interventions should address means to improve uptake of COVID-19 medications. Options to improve communication include discussing medication options at the time of SARS-CoV-2 testing, telehealth visits after positive tests, and education modules targeting clinicians in communities with low COI. Training clinicians to screen for high-risk conditions at the point of testing in the ED, in addition to primary care settings, could also improve uptake by broaching the possibility of medications before discharge. Prioritizing the ED would increase representation of vulnerable patient populations, as demonstrated in our institution’s patient demographics (eAppendix 3 in Supplement 1). However, as evidenced by the majority of patients in the screening group being tested outside of the ED setting, this is unlikely to be the sole explanation for the significant difference in COI, race and ethnicity, underlying diagnosis, and insurance status seen between the 2 groups. Logistical and financial support for families who are interested in receiving medications is also essential. Possibilities include citywide call centers to facilitate scheduling, establishment of temporary mAb infusion centers in low-resourced communities during COVID-19 surges, or transportation vouchers.^23,24^

### Limitations

This study has limitations. One limitation of this study is that the impact on pediatric morbidity could not be estimated, as the number needed to treat (NNT) to prevent hospitalizations in children with high-risk infection is not known from clinical trials of COVID-19 therapeutics. Although the NNT to prevent a pediatric hospitalization is likely larger than the number in adults (16 patients), our findings suggest that screening and outreach in similar high-risk adult populations could be used to reduce COVID-19 hospitalizations and deaths.^21^ Beyond potential to prevent hospitalizations and death due to COVID-19, there is likely a financial benefit to over-resourced health care systems. The resources needed to compensate health care workers’ time and effort to conduct screening plus outreach could be offset by the reduction in costs of COVID-19 associated hospital admission ($1700-$2900 per day).^22^ Another limitation is that we did not assess whether primary care or subspecialty clinicians were aware of SARS-CoV-2 diagnoses in their patients who were identified by screening or were knowledgeable about COVID-19 therapeutics. Furthermore, patients may have been referred to or treated at other unaffiliated institutions. Additionally, there was no cost-effectiveness analysis included in this study. Although the number of hours per week (approximately 4 hours) was estimated for the study, it would be important to explore the potential financial and time burden on various sized medical systems of this intervention with future studies.

## Conclusions

As more testing for COVID-19 is done at home, identifying eligible children for medications with lower COI may become more challenging. More direct measures should be taken to increase access for minoritized groups or individuals with lower COI status to COVID-19 therapeutics. Our data suggest a bias that exists within the referral network to higher COI status patients, non-Hispanic White patients, and patients with private health insurance. Improving access should continue to be a high priority during the COVID-19 pandemic. This experience could also be used to restructure health care outreach and mitigate inequity in responses to future disease outbreaks.

## Notes

Supplement 1. **eAppendix 1. **Eligibility for COVID-19 Treatment at Lurie Children's Hospital **eAppendix 2. **Priority Tiers for Treatment of COVID-19 Monoclonal Antibodies **eAppendix 3. **Characteristics of Patients for all Diagnoses (January 1, 2022, through February 15, 2022) **eAppendix 4. **Types of Clinicians of 51 Patients Referred for COVID-19 Treatment

Supplement 2. Data Sharing Statement.
