# Supplementary material for: Sociodemographic Comparison of Children With High-risk Medical Conditions Referred vs Identified Through Screening Plus Outreach for COVID-19 Therapeutics
Source: JAMA Netw Open. 2022 Dec 28;5(12):e2248671. doi: 10.1001/jamanetworkopen.2022.48671 (PMC9857346; doi:10.1001/jamanetworkopen.2022.48671)
Supplement: Supplement 1. — eAppendix 1. Eligibility for COVID-19 Treatment at Lurie Children's Hospital eAppendix 2. Priority Tiers for Treatment of COVID-19 Monoclonal Antibodies eAppendix 3. Characteristics of Patients for all Diagnoses (January 1, 2022, through February 15, 2022) eAppendix 4. Types of Clinicians of 51 Patients Referred for COVID-19 Treatment [file jamanetwopen-e2248671-s001.pdf]

Supplemental Online Content

Parzen-Johnson S, Sun S, Patel AB, Scardina T, Shah SK, Patel SJ. Sociodemographic comparison of children with high-risk medical conditions referred vs identified through screening plus outreach for COVID-19 therapeutics. *JAMA Netw Open*. 2022;5(12):e2248671. doi:10.1001/jamanetworkopen.2022.48671

eAppendix 1. Eligibility for COVID-19 Treatment at Lurie Children's Hospital
eAppendix 2. Priority Tiers for Treatment of COVID-19 Monoclonal Antibodies
eAppendix 3. Characteristics of Patients for all Diagnoses (January 1, 2022, through February 15, 2022)
eAppendix 4. Types of Clinicians of 51 Patients Referred for COVID-19 Treatment

This supplemental material has been provided by the authors to give readers additional information about their work.

© 2022 Parzen-Johnson S et al. *JAMA Network Open.*

eAppendix 1. Eligibility for COVID-19 Treatment at Lurie Children's Hospital

Patients are eligible if they have of the following clinical presentations AND have an eligible risk factor for progression to severe disease.

Clinical Presentations

- Non-hospitalized children ≥12 years with mild to moderate illness
- Hospitalized children of all ages with mild to moderate illness if reason for admission or continued hospitalization is for concurrent management of chronic conditions*
- Asymptomatic children who test positive for COVID-19**

**Note that patients who require ventilatory/oxygen support over baseline are not eligible.*
***ID service will assess asymptomatic children with positive COVID-19 test for high risk of progression for COVID-19 disease based on chronic condition and date of exposure.*

Risk Factor

- **Neuromuscular or neurological disease with respiratory compromise**
- **Chronic lung disease, including ventilated patients and those on baseline supplemental 02**
- **Sickle cell disease and thalassemia**
- **Congenital or acquired heart disease**[1]
- **High risk asthma**[2]
- **Obesity (BMI> 95th percentile) in children ≥8 years**
- **Genetic or metabolic syndromes**
- **Severe congenital anomalies**
- **An immunocompromising condition or immunosuppressive treatment**[3]

[1]typically includes, but is not limited to, palliated single ventricle/Fontan, chronic cardiac cyanosis (<85%), cardiac dysfunction requiring anti-congestive medications, significant cardiomyopathy requiring medications, pulmonary hypertension, heart transplant.
[2]hospitalization in the ICU in the last 12 months for status asthmaticus; 2 or more hospitalizations in the last 12 months for status asthmaticus, patients on maximal therapy (mid to high dose inhaled corticoid steroids plus second controller) who require systemic steroids.
[3]will be decided on a case-by-case basis due based on degree of immune compromise.

© 2022 Parzen-Johnson S et al. *JAMA Network Open.*

eAppendix 2. Priority Tiers for Treatment of COVID-19 Monoclonal Antibodies

- Tier 1: Patients with one or more of the following conditions:
    - Stem cell transplantation OR CAR-T treatment OR receipt of B-cell depleting agents in the prior 6 months OR receiving immune suppression for GVHD
    - Receipt of solid organ transplant OR treatment of rejection in the prior 6 months
    - Known or suspected primary immunodeficiency (per immunology), including agammaglobulinemia, T cell lymphopenia, requirement of IVIG replacement or antimicrobial prophylaxis
    - Chronic lung disease requiring oxygen OR severe restrictive lung disease including caused by congenital abnormalities or muscular dystrophy
    - Moderately to severely depressed cardiac function OR single ventricle physiology OR complex congenital heart disease, cardiomyopathy or pulmonary HTN requiring heart failure or pulmonary hypertension treatment (medication or mechanical support)
    - Sickle cell disease with 2 or more hospitalizations for pain and/or acute chest syndrome in the last 12 months
    - Obesity BMI >99 %ile

- Tier 2: Patients not in Tier 1 with ≥2 risk factors for progression to severe disease (see Appendix 1).

- Tier 3: Patients with one risk factor for progression to severe disease (see Appendix 1).

Vaccination of patients and family members is strongly recommended to reduce household and community infection, and risk of re-infection.

© 2022 Parzen-Johnson S et al. *JAMA Network Open.*

eAppendix 3: Characteristics of Patients for all Diagnoses (January 1, 2022, through February 15, 2022)

	**Category**	**Total Patients (N = 51,080)**	**Emergency Department Patients (N = 4,854)**
Sex	Female	24,533 (48.0%)	2,274 (46.9%)
Race/Ethnicity	White, Non-Hispanic	20,523 (40.2%)	1,026 (21.1%)
	Black, Non-Hispanic	5,295 (10.4%)	804 (16.6%)
	Hispanic/Latinx	15,479 (30.3%)	2,437 (50.2%)
	Other*	9,647 (18.9%)	575 (11.9%)
Primary Language	English	44,243 (86.6%)	3,915 (80.7%)
Insurance Type	Public	22,684 (44.4%)	3,195 (65.8%)
Composite COI Score	Very High or High	24,679 (48.3%)	1,133 (23.3%)
COI Score (Education)	Very High or High	24,698 (48.4%)	1,163 (24.0%)
COI Score (Health and Environment)	Very High or High	11,484 (22.5%)	353 (7.3%)
COI Score (Social and Economic)	Very High or High	26,833 (52.5%)	1,435 (29.6%)

Note: All proportions were calculated based on non-missing values
(COI = Child Opportunity Index)
*Included mixed race, not identified, mixed/non-Hispanic

© 2022 Parzen-Johnson S et al. *JAMA Network Open.*

eAppendix 4: Types of Clinicians of 51 Patients Referred for COVID-19 Treatment

**Department**	**Number of Referrals**
General Pediatrics	21
Oncology/Stem Cell Transplant	8
Kidney Transplant	5
Cardiac Transplant	4
Pulmonology	3
Gastroenterology	2
Liver Transplant	2
Emergency Department	1
Immunology	1
Infectious Diseases	1
Nephrology	1
Pulmonology/Cardiology	1
Rheumatology	1
Total	51

© 2022 Parzen-Johnson S et al. *JAMA Network Open.*
