# Supplementary material for: Sociodemographic Comparison of Children With High-risk Medical Conditions Referred vs Identified Through Screening Plus Outreach for COVID-19 Therapeutics
Source: JAMA Netw Open. 2022 Dec 28;5(12):e2248671. doi: 10.1001/jamanetworkopen.2022.48671 (PMC9857346; doi:10.1001/jamanetworkopen.2022.48671)
Supplement: Supplement 2. — Data Sharing Statement. [file jamanetwopen-e2248671-s002.pdf]

Data Sharing Statement

Parzen-Johnson. Sociodemographic Comparison of Children With High-Risk Medical Conditions Referred vs Identified Through Screening Plus Outreach for COVID-19 Therapeutics. *JAMA Netw Open*. Published December 28, 2022. doi:10.1001/jamanetworkopen.2022.48671

Data
Data available: No

Additional Information
Explanation for why data not available: Our data includes insurance information, neighborhood data and thus we are unable to share it due to confidentiality concerns. In special circumstances, we are happy to discuss ways to mediate this depending on the request submitted.
